# Impact of Milk Fortification on the Microbiological and Physicochemical Properties of Set-Type Skimmed Yoghurt Using Three Commercial Soluble Prebiotics

**DOI:** 10.3390/foods8060181

**Published:** 2019-05-28

**Authors:** Rui Li, Qi Ding, Xin-Huai Zhao

**Affiliations:** Key Laboratory of Dairy Science, Ministry of Education, Northeast Agricultural University, Harbin 150030, China; lirui09_09@163.com (R.L.); dingqi225@126.com (Q.D.)

**Keywords:** set-type skimmed yoghurt, inulin, iso-malto-oligosaccharides, xylo-oligosaccharides, yoghurt quality

## Abstract

The impact of milk fortification on the microbiological and physicochemical properties of a set-type skimmed yoghurt using three commercial soluble prebiotics (inulin, iso-malto-oligosaccharides, and xylo-oligosaccharides) at either 3 or 5 g/kg was assessed. The three prebiotics had an insignificant impact on yoghurt fermentation because all yoghurt samples had similar titratable acidity and similar pH values after their lactic acid fermentation. Regarding the control yoghurt samples without prebiotics usage, the prebiotics-fortified yoghurt samples showed no difference in their main chemical compositions, hardness, syneresis extent, and apparent viscosity (*p* > 0.05), but had a slightly higher lactic acid content and a viable quantity of starter strains. All yoghurt samples had the same acetic acid content, while propionic and butyric acids were not produced. Yoghurt storage at 4 °C for 21 day gave these yoghurt samples decreased pH values and a viable quantity of starter strains (especially *Lactobacillus delbrueckii* subsp. *bulgaricus*) and unchanged acetic acid; however, it increased lactic acid contents. Overall, prebiotics fortification up to 5 g/kg had a completely insignificant impact on the fermentation and quality attributes of yoghurt samples but could possibly improve the health of consumers due to higher dietary fibers and starter strain populations.

## 1. Introduction

Over the past few decades, yoghurt has become one of the most popular dairy foods in the world due to its diversification in yoghurt-like products such as yoghurt shakes, drinkable yoghurts, yoghurt mousse, yoghurt ice-cream, and others [1]. Yoghurt and yoghurt products are thus considered as healthy foods considering the health benefits of viable lactic acid bacteria (LAB), as LAB can compete with pathogenic bacteria in the digestive system [2]. However, yoghurt and some yoghurt-like products also have some shortcomings from a nutritional point of view. For example, they do not contain enough dietary fibers that have various important physiological functions in the body. Increasing scientific evidence shows that dietary fibers have desired health benefits, such as increasing fecal content, decreasing cholesterol level [3], enhancing the growth of beneficial intestinal bacteria [4], and more importantly, lessening the risk for some diseases like colon cancer [5]. The American Dietetics Association thus suggests the intake of soluble dietary fibers to decrease blood cholesterol levels [6]. Clearly, when dietary fibers (especially soluble and non-digestible saccharides) are added into yoghurt milk, yoghurt and yoghurt-like products provide both probiotic (from starter strains) and prebiotic (from added poly- or oligo-saccharides) functions to consumers. This treatment surely gives yoghurt and yoghurt-like products extra health benefits. Yoghurt with prebiotics fortification might be widely produced in a future market; however, whether these dietary fibers could impact yoghurt fermentation and product quality is not well-verified at present. These potential impacts thus deserve a study as they are very important to yoghurt producers.

Several carbohydrate-based derivatives have prebiotics functions, such as inulin, xylo-oligosaccharides, and iso-malto-oligosaccharides. The three prebiotics have been widely used in processed foods [7,8,9]. Inulin belongs to the fructan compounds: it has a degree of polymerization of 2–60 or more, consists mostly of linear chains of fructosyl moieties linked by β-1,2 linkages, and often ends with a glucosyl unit [10]. Inulin has prebiotic efficiency and can be used as a fat or sugar substitute without any adverse effect on flavor attributes [11]. Inulin therefore can be used in processed foods to improve their nutritional value and stimulate the growth of bifidobacteria [12]. Research has demonstrated the inulin application as a texture modifier in low-fat yoghurt [13], low-fat cheese [14], yoghurt ice cream [7], and fermented camel milk [15]. Adding inulin into camel yoghurt or other set-type yoghurt samples helped to increase the numbers of *Streptococcus thermophilus* and *Lactobacillus delbrueckii* subsp. *bulgaricus* [15,16]. Xylo-oligosaccharides are mainly formed by 2–3 xylose units via β-1,4 linkages [17] and have valued physiological functions such as the maintenance of gastrointestinal health and the reduction of cholesterol level [18]. Xylo-oligosaccharides can promote bifidobacteria growth in the human gut [19] and were also able to inhibit the colon precancerous lesion induced by 1,2-dimethylhydrazine in model animals [20]. In addition, xylo-oligosaccharides have been found more efficient than fructo-oligosaccharides in the promotion of bifidobacteria growth [19]. The most important application of xylo-oligosaccharides is in the field of functional foods, such as soy milk, milk powders, tea, and yoghurt beverages [9]. It was proved that adding up to 3.5% of xylo-oligosaccharides had no impact on the taste and overall acceptability of set-type yoghurt [21]. Iso-malto-oligosaccharides, with α-1,6 linkages in their chemical structures, are mainly composed of iso-maltose, iso-maltotriose, and iso-maltotetraose [22] and exist in several fermented foods such as miso, soya sauce, and sake [23]. Commercial iso-malto-oligosaccharides usually have a degree of polymerization of less than 6 [24]. As a good alternative to classic sweetener table sugar (i.e., commercial sucrose), iso-malto-oligosaccharides are ideal for the formulation of low-calorie foods [25]. Thus, the syrup of iso-malto-oligosaccharides has been used in sponge cakes to replace sucrose [8]. In addition, iso-malto-oligosaccharides have various health benefits, such as activating the immune system, acting as anti-cariogenic agents, and enhancing disease resistance [26].

The mentioned health benefits of inulin, xylo-oligosaccharides, and iso-malto-oligosaccharides support their potential application in dairy foods. However, whether their application in yoghurt processing might impact yoghurt quality needs to be verified. In this study, the three prebiotics were added separately into skimmed milk at two levels, at either 3 or 5 g/kg milk; both did not alter milk compositions significantly. The yoghurt milk was then fermented by a commercial direct vat starter, while the prepared set-type yoghurt samples were stored for different times and measured for their microbiological and physicochemical indices, including acid production, yoghurt hardness, apparent viscosity, syneresis, and viable counts of starter strains using various assaying techniques. Following this, the control yoghurt without added prebiotics was also assessed for these indices. The main aim was to clarify potential impacts of prebiotics fortification on the microbiological and physicochemical properties of set-type skimmed yoghurt.

## 2. Materials and Methods

### 2.1. Material and Chemicals

The skimmed bovine milk powder used for the preparation of skimmed milk was purchased from Fonterra Trading (Shanghai) Co. Ltd. (Shanghai, China), while a direct vat starter (YO-MIX 499) containing *S. thermophilus* and *L. delbrueckii* subsp. *bulgaricus* was provided by Danisco GmbH (Beijing, China). Inulin (purity of 100%), iso-malto-oligosaccharides (purity of 90%), and xylo-oligosaccharides (purity of 95%) were bought from Aladdin Chemistry Co. Ltd. (Shanghai, China), Baolingbao Biology Co. Ltd. (Yucheng, Shandong, China), and Shandong Longlive Biotechnology Co. Ltd. (Yucheng, Shandong, China), respectively. Table sugar (i.e., commercial sucrose) was purchased from a local supermarket in Harbin (Heilongjiang, China). Other chemicals used were of analytical grade. Double-distilled water was used to prepare all solutions.

### 2.2. Sample Preparation

Yoghurt samples were prepared as previously described [27] with slight changes. The skimmed milk (protein content about 32 g/kg) was mixed with the table sugar (60 g/kg milk), and then used to prepare a control yoghurt (Yoghurt I). The skimmed milk was also mixed with table sugar (60 g/kg milk) and one of the three prebiotics at two levels (3 or 5 g/kg milk), and then used to prepare six prebiotics-fortified yoghurt samples (Yoghurts II−VII). After mixing the skimmed milk with table sugar and the prebiotics, the yoghurt milk samples were all heated at 95 °C for 15 min, cooled to about 42 °C, and inoculated with the starter at 0.06 g/kg milk (recommended by the starter supplier). After that, the milk samples were poured into 100 mL glass containers of 60 mm diameter under aseptic condition and fermented at 42 °C for about 5 h until the value of pH fell to near 4.5. The yoghurt samples were all placed in a refrigerator at 4 °C for 1, 7, and 21 day, respectively, and selected randomly to assess their physicochemical and microbiological indices.

### 2.3. Chemical and Rheological Assays

Protein, total solids contents, and titratable acidity of the yoghurt samples were assayed using the respective Kjeldahl, oven-drying, and titration methods [28]. Values of pH were monitored using a pH meter (Mettler, Toledo, DELTA-320, pH, Shanghai, China).

Apparent viscosity was measured at 25 °C as per the studies [29,30], using a Bohlin Gemini II Rheometer (Malvern Instruments Limited, Worcestershire, UK) and a cone-plate geometry (40 mm diameter, 4° angle, 150 μm gap). The yoghurt samples were equilibrated to 25 °C and stirred gently by hand rotating 10 times with a tablespoon to ensure a visually homogeneous state, followed by an increasing sweep at the Rheometer using shear rates ranging from 0.1 to 10 s^−1^.

Thixotropic behaviors of the yoghurt samples were measured as previously described [29]. The samples were loaded on the plate, and sheared at 500 s^−1^ for 1 min. The flow curves were evaluated by increasing shear rates (0.1–100 s^−1^) within 30 min and then decreasing shear rates (100–0.1 s^−1^) within 30 min. The area of hysteresis loop between upward and downward flow curves was calculated by the software in the Rheometer.

### 2.4. Assays of Hardness and Syneresis

A penetration test using the texturometer (Model TA-XT2i, Stable Micro Systems Ltd., Surry, UK) with a 5 kg load cell was applied to assay yoghurt hardness [31,32]. The yoghurt samples (60 mm diameter × 60 mm height) held in the glass containers were equilibrated to 20 °C. A 35 mm diameter cylindrical probe and two cycles were applied at a constant cross-head velocity of 1 mm/s and a surface trigger of 10 g to a fixed sample depth of 30 mm. Hardness values were calculated by the XT.RA Dimension software version 3.7 (Stable Micro Systems Ltd., Surry, UK).

Syneresis of the yoghurt samples were assayed using a centrifugation method [30]. The yoghurt milk about 20 mL was fermented at 42 °C for about 5 h in 50 mL centrifugation tubes sterilized before, stored at 4 °C for 24 h and then centrifuged at 640× *g* for 10 min at 25 °C. The supernatant was poured and weighed. Syneresis was calculated as the percentage value of the collected supernatant to original yoghurt sample on a weight basis.

### 2.5. Assays of Lactic Acid and Other Three Organic Acids

The yoghurt samples were centrifuged at 4200× *g* for 10 min to collect their supernatants. The supernatants of 1 mL were mixed with 0.5 mL 10.6% potassium ferrocyanide, 0.5 mL 21.9% zinc acetate, and 3 mL water, left stand for 30 min, and then centrifuged at 4200× *g* for 10 min. The supernatants were collected, properly diluted, and used to detect lactic acid contents as previously described [33]. The measurement was carried out at 565 nm using a UV-2401 spectrophotometer (Shimadzu Co. Ltd., Kyoto, Japan).

The yoghurt samples were centrifuged at 4200× *g* for 10 min. The obtained supernatants of 1 mL were mixed with absolute ethanol of 2.4 mL in test tubes. The generated mixtures of 0.85 mL were transferred to low borosilicate glass ampoules, followed by the addition of 0.1 mL concentrated sulfuric acid and 1 mL hexane. The ampoules were sealed by a blast burner, heated in a water bath of 60 °C for 1 h, cooled to 20 °C, and then mixed vigorously for 5 min. The upper hexane phases were passed through syringe membrane filters (0.45 μm Nylon 6, Tianjin Navigator Lab Instrument Co. Ltd., Tianjin, China), and then detected by gas chromatography (GC) method [34] to obtain the contents of acetic, propionic, and butyric acids. Standard acid solutions were used to generate standard curves for this measurement.

GC analysis was performed using an Agilent 7890 Gas Chromatography system (Agilent Technologies, Santa Clara, CA, USA) fitted with a flame ionization detector and a HP-5 capillary column (30 m × 0.32 mm). Nitrogen as the carrier gas was delivered at a rate of 1.0 mL/min and a split ratio of 40:1. The initial oven temperature was 30 °C, which was maintained for 3.5 min, raised to 40 °C at 8 °C/min, and then increased to 150 °C at 15 °C/min. Injector and detector temperatures were set at 200 °C and 220 °C, respectively. A sample of 1 μL was used in this analysis.

### 2.6. Determination of Viable Starter Strains

The determination was carried out as per the study [35] with slight changes. M17 and MRS agar media were used for *S. thermophilus* and *L. delbrueckii* subsp. *bulgaricus* counting, respectively. Yoghurt samples (1 mL) were mixed with 0.9% sterilized NaCl solution (9 mL), and then homogenized. Decimal dilution of the homogenates was conducted. Aliquots of the diluted homogenates (0.1 mL) were inoculated over the surfaces of the media (about 20 mL) in the plates. After that, all plates were incubated at 37 °C for (48 ± 2) h. The M17 and MRS agar media were cultured under aerobic and anaerobic conditions, respectively. The populations of different species were calculated from the developed colony-forming units. Colonies were confirmed as either *Lactobacillus* or *Streptococcus* by both gram staining and microscopic observation.

### 2.7. Statistical Analysis

All experiments and analyses in this study were carried at least three times, and all data were reported as means or means ± standard deviations. The differences between means of multiple groups were analyzed by the one-way analysis of variance (ANOVA) with Duncan’s multiple range tests using the SPSS 16.0 software (SPSS Inc., Chicago, IL, USA).

## 3. Results and Discussion

### 3.1. Effect of Prebiotics Fortification on the Acidity of Yoghurt Samples

The study indicated that all prepared yoghurt samples had similar protein (from 29.6 to 30.8 g/kg) and total solids (from 150.1 to 154.4 g/kg) contents. Prebiotics fortification of yoghurt milk did not impact these two indices (*p* > 0.05). All yoghurt samples after storage of one day had slight differences in titratable acidity but showed similar pH values (*p* > 0.05) (Table 1). In detail, Yoghurts II−VII showed very close titratable acidity and pH values (0.79–0.84% versus 0.81%, and pH 4.40–4.44 versus pH 4.42), compared with Yoghurt I. Furthermore, prebiotics fortification at 3 or 5 g/kg milk did not lead to significant differences in titratable acidity and pH values. This fact means that prebiotics fortification had very little impact on acid production during yoghurt fermentation. Similar results were found in other studies. Inulin addition had no influence on pH values of both low-fat and whole-fat yoghurts [13], while inulin used as fat replacer did not affect the pH value and titratable acidity of the set-type yoghurt [36]. These two studies proved that prebiotics fortification had no impact on the acid production during yoghurt fermentation. However, opposite phenomena have also been observed in past studies; for example, it was indicated that the yoghurt samples fortified with respective inulin, short-chain inulin, and lactulose showed higher or lower pH values than the control yoghurt sample [12,37].

All prepared yoghurt samples showed significant increases in titratable acidity but significant decreases in pH values during storage for 21 day (*p* < 0.05) (Table 1) as a result of yoghurt post-acidification. When these yoghurt samples were stored for 7 day, the values of titratable acidity were increased from 0.79–0.84% to 0.88–0.91% (*p* < 0.05), while pH values were decreased from 4.39–4.44 to 4.24–4.28 (*p* < 0.05). The 21-day storage led to further increases (0.93–0.96%) in titratable acidity and further decreases (4.11–4.19) in pH values. In general, Yoghurt I–VII stored for 7 or 21 day had close values in titratable acidity and pH, demonstrating that prebiotics fortification did not promote or inhibit yoghurt post-acidification. In other words, prebiotics fortification had an insignificant impact on the quality of the stored yoghurt samples. Moreover, the prebiotics fortification level also showed an insignificant effect on yoghurt post-acidification, because Yoghurts II, IV, and VI showed similar index values to Yoghurts III, V, and VII, respectively. Usually, the increased titratable acidity and decreased pH value of the stored yoghurt is caused by the post-acidification; for example, two previous studies had found that pH values of yoghurt samples were decreased during storage for 14 day [12,38]. A similar pH decrease was also detected in the low-fat yoghurt stored for 15 day [36]. The present results were thus consistent with three reported studies.

### 3.2. Effect of Prebiotics Fortification on Organic Acid Contents

Four short-chain organic acids (acetic, propionic, butyric, and lactic acids) were measured when the yoghurt samples were stored at 4 °C for 1, 7, and 21 day, respectively. The obtained results are shown in Table 2.

Both propionic and butyric acids existed in yoghurt samples but were detected at very low levels. In total, the detected contents of propionic and butyric acids were less than the respective detection limits of 59 and 35 mg/kg in this study. These two acids were thus considered as not to be produced in the yoghurt samples. That is, prebiotics fortification did not result in extra production of the two acids in Yoghurts II–VII. Regarding acetic acid, it was detected in all yoghurt samples; however, the contents were measured in very similar and stable amounts (0.09–0.10 g/kg), even if the yoghurt samples were stored for 21 day. Prebiotics fortification thereby did not change acetic acid production in the yoghurt samples (*p* > 0.05). However, both prebiotics fortification and storage time showed a clear impact on lactic acid production. In general, prebiotics fortification resulted in Yoghurts II–VII stored for 7 or 21 day with lactic acid contents higher than Yoghurt I (*p* < 0.05), and longer storage time always led to higher lactic acid contents (*p* < 0.05). After storage of 1 or 7 day, Yoghurts II–VII had lactic acid contents of 9.20–10.78 g/kg, whilst Yoghurts I had lactic acid contents of 8.57–9.74 g/kg. Long-time storage (i.e., 21 day) endowed Yoghurts II–VII with enhanced lactic acid contents of 12.72–13.06 g/kg, whilst lactic acid content of Yoghurt I was only enhanced to 10.68 g/kg. Lactic acid contents of Yoghurts II–VII with short storage time (1 or 7 day) were observed to be dependent on prebiotics addition levels. It can be seen from these data in Table 2 that when the yoghurt samples were stored for 1 or 7 day, higher prebiotic addition (5 g/kg) led to lower lactic acid production (Yoghurt II versus Yoghurt III, or Yoghurt IV versus Yoghurt V, or Yoghurt VI versus Yoghurt VII). However, with longer storage of 21 day, this effect (prebiotics addition levels) on lactic acid production was no longer observed in Yoghurts II–VII; on the contrary, the higher prebiotics level of 5 g/kg would lead to higher lactic acid production in the yoghurt samples. These phenomena are not discussed or revealed in the present study but need a detailed investigation in future.

Lactic acid production during yoghurt storage has been verified as the results of further lactose hydrolysis and fermentation [38]. The yoghurt samples fortified with lactulose or inulin during storage of 14 day showed enhanced lactic acid content from 9.9–11.8 g/kg (zero day) to 13.3–15.8 g/kg (14 day), but inulin or lactulose itself had no impact on lactic acid yield [12]. During storage for 28 day, the set-type yoghurt samples had increased the lactic acid content; however, acetic acid content was constant [17]. It was also been found that addition of inulin or high amylose maize starch had an insignificant effect on the production of propionic and butyric acids in the set-type yoghurt samples [17]. These mentioned studies showed conclusions similar to the present study. On the other hand, the production of acetic and lactic acids in the set-type yoghurt using individual culture (*L. acidophilus* or *L. casei*) was enhanced by the addition of inulin and high amylose maize starch, and inulin was found more capable of yielding higher acetic and lactic acid contents [17]. The present results proved that prebiotics fortification had no impact on the yields of acetic, propionic, and butyric acids in yoghurt samples, but only increased lactic acid production by 7–11% at 1 day, 8–11% at 7 day, or 19–22% at 21 day. This suggests that prebiotics fortification in the yoghurt samples overall had a slight impact on the production of the four organic acids.

### 3.3. Effect of Prebiotics Fortification on Texture of Yoghurt Samples

**Figure 1:**
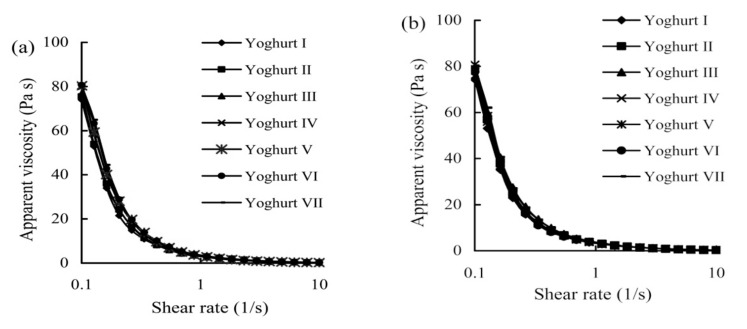
Apparent viscosity values of Yoghurts I–VII stored for 1 day (**a**) or 21 day (**b**). Yoghurt I is the control yoghurt without prebiotics fortification. Yoghurts II, IV, and VI are the yoghurt samples fortified with inulin, iso-malto-oligosaccharides, and xylo-oligosaccharides of 3 g/kg milk, while Yoghurts III, V, and VII are the yoghurt samples fortified with inulin, iso-malto-oligosaccharides, and xylo-oligosaccharides of 5 g/kg milk, respectively.

Based on these obtained data (Table 3 and Figure 1), all yoghurt samples with same storage time overall had a similar hardness, syneresis, apparent viscosity, and hysteresis loop areas, because these measured index values were insignificantly different (*p* > 0.05). The three prebiotics at 3–5 g/kg exerted little impact on total solids contents of Yoghurts II–VII. All yoghurt samples reasonably had similar values of hardness and apparent viscosity. Prebiotics fortification level overall had an insignificant impact on these indices. Long-time storage (21 day) mostly resulted in yoghurt samples with slight decreases in hardness and hysteresis loop areas but slight increases in syneresis. However, further data comparison indicated that yoghurt samples stored 1 day overall had index values close to those stored for 21 day (*p* < 0.05), namely, both prebiotics fortification and storage time had an insignificant impact on yoghurt texture.

Addition of food stabilizer can improve yoghurt texture [39]. However, addition of some dietary fibers results in insignificant or significant influence on yoghurt texture. The low-fat yoghurt fortified with inulin of 10–30 g/kg had no impact yoghurt texture [36]. Application of the dietary fibers from four plant resources at 13 g/kg also showed no impact on both rheological and sensory properties of yoghurt samples [2]. These two studies thus provided support to the present results. On the contrary, some studies had used higher levels of dietary fibers in yoghurt processing, resulting in improved yoghurt texture. For example, addition of inulin up to 20 g/kg reduced yoghurt syneresis [13], while fortification of yoghurt with long-chain inulin of 15 g/kg led to lower syneresis but better texture [37]. At the same time, a higher level of prebiotics addition also can confer yoghurt with enhanced rheological properties. It was observed that inulin added at higher levels (10–40 g/kg) in the low-fat yoghurt resulted in a significant increase in viscosity [13], while using beta-glucan (more than 10 g/kg) in the low-fat yoghurt brought about increased viscosity [40]. This study used three prebiotics at lower levels (3–5 g/kg). The three prebiotics therefore showed little impact on the yoghurt texture. Moreover, the storage period can impact the yoghurt quality. Two past studies found that yoghurt samples during storage for 14 day had increased syneresis values [41] and worse texture [37]. The present study also found slightly increased syneresis values for the yoghurt samples with a storage time of 21 day.

### 3.4. Effect of Prebiotics Fortification on Survival of Starter Strains

The results listed in Table 4 show the changes in viable counts of two starter strains (*S. thermophilus* and *L. delbrueckii* subsp. *bulgaricus*) in Yoghurts I–VII at three storage periods. On the first day, Yoghurt I (without prebiotics) contained *S. thermophilus* and *L. delbrueckii* subsp. *bulgaricus* at about 3.7 × 10^8^ and 2.0 × 10^8^ cfu/mL, while Yoghurts II–VII showed slight higher viable counts of *S. thermophilus* ((4.1–5.2) × 10^8^ cfu/mL) and *L. delbrueckii* subsp. *bulgaricus* ((2.8–3.7) × 10^8^ cfu/mL). On day 7, Yoghurt I showed somewhat increased viable counts of *S. thermophilus* and *L. delbrueckii* subsp. *bulgaricus* (4.0 × 10^8^ and 2.6 × 10^8^ cfu/mL, respectively), while Yoghurts II–VII were measured with decreased counts of the two strains, as the respective *S. thermophilus* and *L. delbrueckii* subsp. *bulgaricus* counts were (3.9–5.2) × 10^8^ and (1.6–2.7) × 10^8^ cfu/mL. On day 21, viable counts of *S. thermophilus* and *L. delbrueckii* subsp. *bulgaricus* in Yoghurt I were 3.9 × 10^8^ and 1.1 × 10^8^ cfu/mL, respectively. However, yoghurts II–VII had viable counts of *S. thermophilus* and *L. delbrueckii* subsp. *bulgaricus* of (3.5–4.9) × 10^8^ and (0.7–2.0) × 10^8^ cfu/mL, respectively. Clearly, long-time storage (21 day) mostly led to decreased viable counts of *L. delbrueckii* subsp. *bulgaricus* (*p* < 0.05); however, viable counts of *S. thermophilus* mostly showed an insignificant decreasing trend (*p* > 0.05). Compared with those with prebiotics fortification of 3 g/kg milk, the yoghurt samples with prebiotics fortification of 5 g/kg milk mostly showed close viable counts of the two strains at these time points, suggesting that the used prebiotics levels had no influence on starter survival. However, compared with Yoghurt I, prebiotics fortification led to somewhat higher counts of the two strains in the fortified yoghurt samples (*p* < 0.05), demonstrating potential growth stimulation of the three prebiotics on the two strains. Higher counts of two starter strains in Yoghurts II–VII led to stronger fermentation. Yoghurts II–VII thus had higher lactic acid contents, compared with Yoghurt I (as these data listed in Table 2).

Donkor and coauthors found that addition of inulin to yoghurt increased the viable counts of both *S. thermophilus* and *L. delbrueckii* subsp. *bulgaricus* [16]. When inulin was added into camel milk at 60 g/L, in comparison with the control yoghurt, the prepared yoghurt had a one-fold increase in both *S. thermophilus* and *L. delbrueckii* subsp. *bulgaricus* [15]. The two studies thus proved that some prebiotics had growth stimulation on starter strains of yoghurt. However, yoghurt storage can lead to decreased counts of starter strains, because these strains are mostly susceptible to the acidic environment of yoghurt [42]. It was thereby evident that yoghurt storage at 4 °C for 35 day led to decreased counts of both *S. thermophilus* and *L. delbrueckii* subsp. *bulgaricus* [35], and a frozen yoghurt sample fortified with inulin also showed decreased probiotics counts during storage [43]. These mentioned results supported the present results. Based on the well-known health significance of dietary fibers and LAB, this study showed that prebiotic fortification could confer yoghurt products with higher soluble dietary fiber content and LAB populations, which should be considered to improve the health of consumers.

## 4. Conclusions

Based on the obtained chemical, mechanical, and microbiological results, the effect of milk fortification with three soluble prebiotics inulin, iso-malto-oligosaccharides, and xylo-oligosaccharides on several quality attributes of set-type skimmed yoghurt was verified. In general, prebiotics fortification did not delay yoghurt fermentation or change the production of acetic, propionic, and butyric acids. Prebiotics fortification also had very little influence on yoghurt hardness and syneresis, but in some extents could increase lactic acid content and promote the growth of two starter strains to enhance viable starter counts. It is concluded that prebiotics fortification up to 5 g/kg overall had an insignificant impact on microbiological and physicochemical properties of the set-type yoghurt samples. However, prebiotics fortification is beneficial to yoghurt processing, as yoghurt samples therefore have a higher soluble dietary fiber content and starter strain populations.

## Figures and Tables

**Table 1:** Titratable acidity (lactic acid %) and pH values of Yoghurts I–VII during their storage at 4 °C.

Samples	Titratable Acidity			pH Values		
	1 day	7 day	21 day	1 day	7 day	21 day
Yoghurt I	0.81 ± 0.01 ^bA^	0.90 ± 0.01 ^cB^	0.93 ± 0.01 ^aC^	4.42 ± 0.02 ^aC^	4.28 ± 0.01 ^aB^	4.16 ± 0.02 ^aA^
Yoghurt II	0.84 ± 0.01 ^cA^	0.91 ± 0.01 ^dB^	0.94 ± 0.01 ^abC^	4.41 ± 0.03 ^aC^	4.24 ± 0.02 ^aB^	4.18 ± 0.04 ^aA^
Yoghurt III	0.81 ± 0.01 ^bA^	0.89 ± 0.01 ^bB^	0.96 ± 0.01 ^bC^	4.44 ± 0.01 ^aC^	4.28 ± 0.01 ^aB^	4.16 ± 0.01 ^aA^
Yoghurt IV	0.80 ± 0.01 ^abA^	0.89 ± 0.01 ^bB^	0.93 ± 0.01 ^aC^	4.41 ± 0.01 ^aC^	4.28 ± 0.03 ^aB^	4.13 ± 0.02 ^aA^
Yoghurt V	0.83 ± 0.01 ^cA^	0.88 ± 0.01 ^aB^	0.94 ± 0.01 ^abC^	4.40 ± 0.03 ^aC^	4.28 ± 0.01 ^aB^	4.16 ± 0.04 ^aA^
Yoghurt VI	0.79 ± 0.01 ^aA^	0.90 ± 0.01 ^cB^	0.94 ± 0.01 ^abC^	4.39 ± 0.03 ^aC^	4.28 ± 0.04 ^aB^	4.11 ± 0.01 ^aA^
Yoghurt VII	0.81 ± 0.01 ^bA^	0.91 ± 0.01 ^dB^	0.94 ± 0.01 ^aC^	4.41 ± 0.05 ^aC^	4.25 ± 0.02 ^aB^	4.19 ± 0.08 ^aA^

Yoghurt I is the control yoghurt without prebiotics fortification. Yoghurts II, IV, and VI are yoghurt samples fortified with inulin, iso-malto-oligosaccharides, and xylo-oligosaccharides of 3 g/kg milk, while Yoghurts III, V, and VII are yoghurt samples fortified with inulin, iso-malto-oligosaccharides, and xylo-oligosaccharides of 5 g/kg milk, respectively. ^a–c^ Means ± standard deviations in the same column with different superscript lowercase letters differ significantly (*p* < 0.05). ^A–C^ Means ± standard deviations in the same row with different superscript uppercase letters differ significantly (*p* < 0.05).

**Table 2:** Lactic and acetic acids contents (g/kg) of Yoghurts I−VII during their storage at 4 °C.

Samples	Lactic Acid			Acetic Acid		
	1 day	7 day	21 day	1 day	7 day	21 day
Yoghurt I	8.57 ± 0.28 ^aA^	9.74 ± 0.30 ^aB^	10.68 ± 0.27 ^aC^	0.09 ± 0.01 ^aA^	0.09 ± 0.01 ^aA^	0.10 ± 0.01 ^aB^
Yoghurt II	9.55 ± 0.01 ^bA^	10.72 ± 0.17 ^bB^	12.72 ± 0.01 ^bC^	0.09 ± 0.01 ^aA^	0.10 ± 0.01 ^bB^	0.10 ± 0.01 ^aB^
Yoghurt III	9.20 ± 0.01 ^bA^	10.65 ± 0.54 ^bB^	12.94 ± 0.05 ^bC^	0.09 ± 0.01 ^aA^	0.09 ± 0.01 ^aA^	0.10 ± 0.01 ^aB^
Yoghurt IV	9.53 ± 0.25 ^bA^	10.65 ± 0.21 ^bB^	12.92 ± 0.13 ^bC^	0.09 ± 0.01 ^aA^	0.09 ± 0.01 ^aA^	0.10 ± 0.01 ^aB^
Yoghurt V	9.26 ± 0.20 ^bA^	10.52 ± 0.02 ^bB^	13.06 ± 0.21 ^bC^	0.09 ± 0.01 ^aA^	0.09 ± 0.01 ^aA^	0.10 ± 0.01 ^aB^
Yoghurt VI	8.61 ± 0.31 ^aA^	10.78 ± 0.10 ^bB^	12.95 ± 0.53 ^bC^	0.09 ± 0.01 ^aA^	0.10 ± 0.01 ^bB^	0.10 ± 0.01 ^aB^
Yoghurt VII	8.49 ± 0.63 ^aA^	10.68 ± 0.13 ^bB^	13.02 ± 0.07 ^bC^	0.09 ± 0.01 ^aA^	0.10 ± 0.01 ^bB^	0.10 ± 0.01 ^aB^

^a–c^ Means ± standard deviations in the same column with different superscript lowercase letters differ significantly (*p* < 0.05). ^A–C^ Means ± standard deviations in the same row with different superscript uppercase letters differ significantly (*p* < 0.05).

**Table 3:** Hardness and syneresis of Yoghurts I–VII after storage of 1 and 21 day at 4 °C.

Samples	Storage Time of 1 day			Storage Time of 21 day		
	Hardness (g)	Syneresis (%)	Hysteresis Loop Area	Hardness (g)	Syneresis (%)	Hysteresis Loop Area
Yoghurt I	139.6 ± 2.7 ^a^	30.9 ± 1.4 ^a^	209.4 ± 9.0 ^a^	138.2 ± 3.2 ^a^	31.8 ± 0.1 ^a^	209.0 ± 6.0 ^a^
Yoghurt II	141.7 ± 4.0 ^a^	30.0 ± 1.4 ^a^	215.2 ± 6.3 ^a^	139.4 ± 4.8 ^a^	32.1 ± 0.7 ^a^	210.8 ± 6.3 ^a^
Yoghurt III	138.2 ± 1.8 ^a^	30.4 ± 0.8 ^a^	219.3 ± 8.8 ^a^	139.4 ± 1.8 ^a^	32.8 ± 0.3 ^a^	210.7 ± 9.8 ^a^
Yoghurt IV	141.2 ± 2.9 ^a^	29.9 ± 0.9 ^a^	211.2 ± 7.3 ^a^	138.7 ± 3.3 ^a^	32.0 ± 0.2 ^a^	208.2 ± 5.2 ^a^
Yoghurt V	139.5 ± 1.7 ^a^	30.2 ± 1.2 ^a^	212.5 ± 2.2 ^a^	139.1 ± 3.9 ^a^	31.7 ± 0.6 ^a^	210.5 ± 6.1 ^a^
Yoghurt VI	138.1 ± 0.5 ^a^	30.6 ± 1.4 ^a^	214.4 ± 4.3 ^a^	139.1 ± 3.4 ^a^	32.6 ± 0.7 ^a^	211.9 ± 8.3 ^a^
Yoghurt VII	138.5 ± 1.5 ^a^	30.3 ± 1.2 ^a^	219.1 ± 10.0 ^a^	138.4 ± 3.3 ^a^	32.8 ± 1.3 ^a^	211.8 ± 9.7 ^a^

^a–c^ Means ± standard deviations in the same column with different superscript lowercase letters differ significantly (*p* < 0.05).

**Table 4:** Counting changes of two viable starter strains in Yoghurts I–VII during their storage of 21 day at 4 °C.

Samples	*S. thermophilus* (10^8^ cfu/mL)			*L. delbrueckii* subsp. *bulgaricus* (10^8^ cfu/mL)		
	1 day	7 day	21 day	1 day	7 day	21 day
Yoghurt I	3.7 ± 0.3 ^aA^	4.0 ± 0.1 ^abA^	3.9 ± 0.2 ^bA^	2.0 ± 0.5 ^aB^	2.6 ± 0.2 ^abB^	1.1 ± 0.2 ^abA^
Yoghurt II	5.1 ± 0.4 ^cA^	5.1 ± 0.4 ^cA^	4.8 ± 0.1 ^cA^	3.3 ± 0.6 ^bB^	1.6 ± 0.6 ^aA^	1.0 ± 0.1 ^abA^
Yoghurt III	5.2 ± 0.3 ^cA^	5.2 ± 0.3 ^cA^	4.8 ± 0.3 ^cA^	3.0 ± 0.3 ^abC^	1.9 ± 0.2 ^abB^	0.9 ± 0.4 ^abA^
Yoghurt IV	5.2 ± 0.3 ^cA^	5.2 ± 0.5 ^cA^	4.9 ± 0.3 ^cA^	2.9 ± 0.5 ^abB^	1.6 ± 0.5 ^aAB^	0.7 ± 0.3 ^aA^
Yoghurt V	4.4 ± 0.4 ^bA^	4.5 ± 0.1 ^bA^	4.1 ± 0.2 ^bA^	2.8 ± 0.7 ^abB^	2.0 ± 0.5 ^abAB^	1.3 ± 0.1 ^bA^
Yoghurt VI	4.1 ± 0.4 ^abB^	3.9 ± 0.1 ^aB^	3.5 ± 0.1 ^aA^	3.7 ± 0.5 ^bB^	2.5 ± 0.4 ^abA^	2.0 ± 0.1 ^cA^
Yoghurt VII	4.2 ± 0.2 ^abB^	4.3 ± 0.2 ^abB^	3.8 ± 0.1 ^abA^	3.6 ± 0.8 ^bB^	2.7 ± 0.4b ^AB^	1.9 ± 0.3 ^cA^

^a–c^ Means ± standard deviations in the same column with different superscript lowercase letters differ significantly (*p* < 0.05). ^A–C^ Means ± standard deviations in the same row with different superscript uppercase letters differ significantly (*p* < 0.05).
